# Quantitative analysis of internal components of the human crystalline lens during accommodation in adults

**DOI:** 10.1038/s41598-021-86007-6

**Published:** 2021-03-23

**Authors:** Yan Xiang, Ting Fu, Qiongfang Xu, Wei Chen, Zhiqi Chen, Jinming Guo, Chaohua Deng, Anne Manyande, Ping Wang, Hong Zhang, Xuebi Tian, Junming Wang

**Affiliations:** 1 Department of Ophthalmology, Tongji Hospital, Tongji Medical College, Huazhong University of Science and Technology, Jiefangroad1095#, Wuhan, 430030 Hubei China; 2 Department of Anesthesiology, Tongji Hospital, Tongji Medical College, Huazhong University of Science and Technology, Jiefangroad1095#, Wuhan, 430030 Hubei China; 3 School of Human and Social Sciences, University of West London, London, UK

**Keywords:** Preclinical research, Anatomy

## Abstract

To quantitatively analyze changes in the inner components of the human crystalline lens during accommodation in adults. Eyes of 23 subjects were sequentially examined using CASIA2 Optical Coherence Tomography under 0D, − 3D and − 6D accommodation states. The anterior chamber depth (ACD), anterior and posterior crystalline lens radius of the curvature (ALRC and PLRC) were obtained using built-in software. The lens thickness (LT), lenticular nucleus thickness (NT), anterior cortex thickness (ACT), posterior cortex thickness (PCT), anterior and posterior lenticular nucleus radius of the curvature (ANRC and PNRC), anterior and posterior lenticular nucleus vertex (ANV and PNV) were quantified manually with the Image-pro plus software. During accommodation, the ACD became significantly shallower and LT significantly increased. For changes in the lens, the ALRC decreased by an average magnitude (related to accommodative stimuli) 0.44 mm/D, and PLRC decreased 0.09 mm/D. There was no difference for the ACT and PCT in different accommodation states. For lenticular nucleus response, NT increased on average by 30 μm/D. Both the ANRC and PNRC decreased on average by 212 μm/D and 115 μm/D respectively. The ANV moved forward on average by 0.07 mm under − 3D accommodative stimuli and 0.16 mm for − 6D. However, there was no statistically significant difference between different accommodation states in the PNV movement. Under accommodation stimulation, lens thickness changed mainly due to the lenticular nucleus, but not the cortex. For the lenticular nucleus, both the ANRC and PNRC decreased and ANRC changed the most. The anterior surface of the nucleus moved forward while the posterior surface of the nucleus moved backward but only slightly.

## Introduction

Accommodation is the ability to provide clear vision during near tasks by increasing the refractive power mainly through crystal lens changes. As accommodation ability decreases and the crystalline hardens, presbyopia often occurs in middle age. And the change in stiffness of the lens material is thought to be responsible for presbyopia. Recently, interest has focused on developing surgical treatments that restore accommodation, including lens photodisruption^1^ and lens refilling^2–4^. To fully understand the mechanism of accommodation and clarify the function of the internal structure of the lens during accommodation is very important for developing effective therapeutic strategies. In particular, the nuclear core and the cortex of the lens have distinct different properties^5^ and many details about the dynamic optomechanical response of the internal structure of the lens under accommodation stimuli have yet to be quantified.

Technologies of slit-lamp photography^6–8^, Scheimpflug photography^9^ were used to measure the change in the internal structure of the lens during accommodation. However, there are several limitations to these technologies. Firstly, stimulation was on the fellow eye but not directly on the testing eye in these two testing modalities. Secondly, to avoid light effects on the pupil, lens images were obtained on pupil pharmacological dilation, but not on physiological status^8^. Thirdly, images from these early techniques presented relatively lower resolution than modern Optical coherence tomography (OCT) techniques^10^.

OCT is a low-coherence, interferometry-based imaging modality that provides a high-resolution, noncontact, noninvasive cross sectional image of the anterior segment^11^. The CASIA 2 OCT (Tomey, Nagoya, Japan) can produce a higher sensitivity for depth, axial and transverse spatial resolution with lateral dimension measuring 16 mm and axial depth 13 mm. This enables data to be obtained from the cornea to the posterior lens in one image and identifying the capsule, cortex and nucleus of lens. Thus, it is the excellent technology for imaging the internal component of crystalline lens during accommodation in vivo. Further, its built-in programs provide the required accommodative stimulation and the individual precise refractive error correction including correcting astigmatism. Previous research^12–14^ has shown that CASIA2 OCT can provide good reproducible measurements of lens biometry in both static and dynamic states. In addition, CASIA2 can correct the optical distortion produced by the cornea, aqueous humor and lens with a homogeneous refractive index included in their built-in program^13,14^, which can obtain accurate anterior and posterior lens component shapes. Therefore, the purpose of the present investigation is to measure changes in the internal component of the crystalline eye lens at different accommodation states with the CASIA2 OCT.

## Results

A total of 23 adults aged between 30 and 40 years old were recruited. One was excluded due to low accommodation amplitude of less than − 6D. Thus, a total of 22 subjects (12 males; 10 female) were eventually included in the analyses. The mean values for various variables across subjects were as follows: age, 34.0 ± 2.2 years; refraction, – 1.6 ± 0.5 diopters; intraocular pressure, 16.6 ± 2.6 mmHg; and amplitude of accommodation, 9.1 ± 2.1 diopters. During accommodation, neither angle to angle distance (ATA) nor corneal thickness (CT) changed, indicating that movement of eye between scans is negligible (F_ATA_ = 2.58, P = 0.11; F_CT_ = 1.35, P = 0.27).

### The changes of the lens shape during accommodation

**Figure 1:**
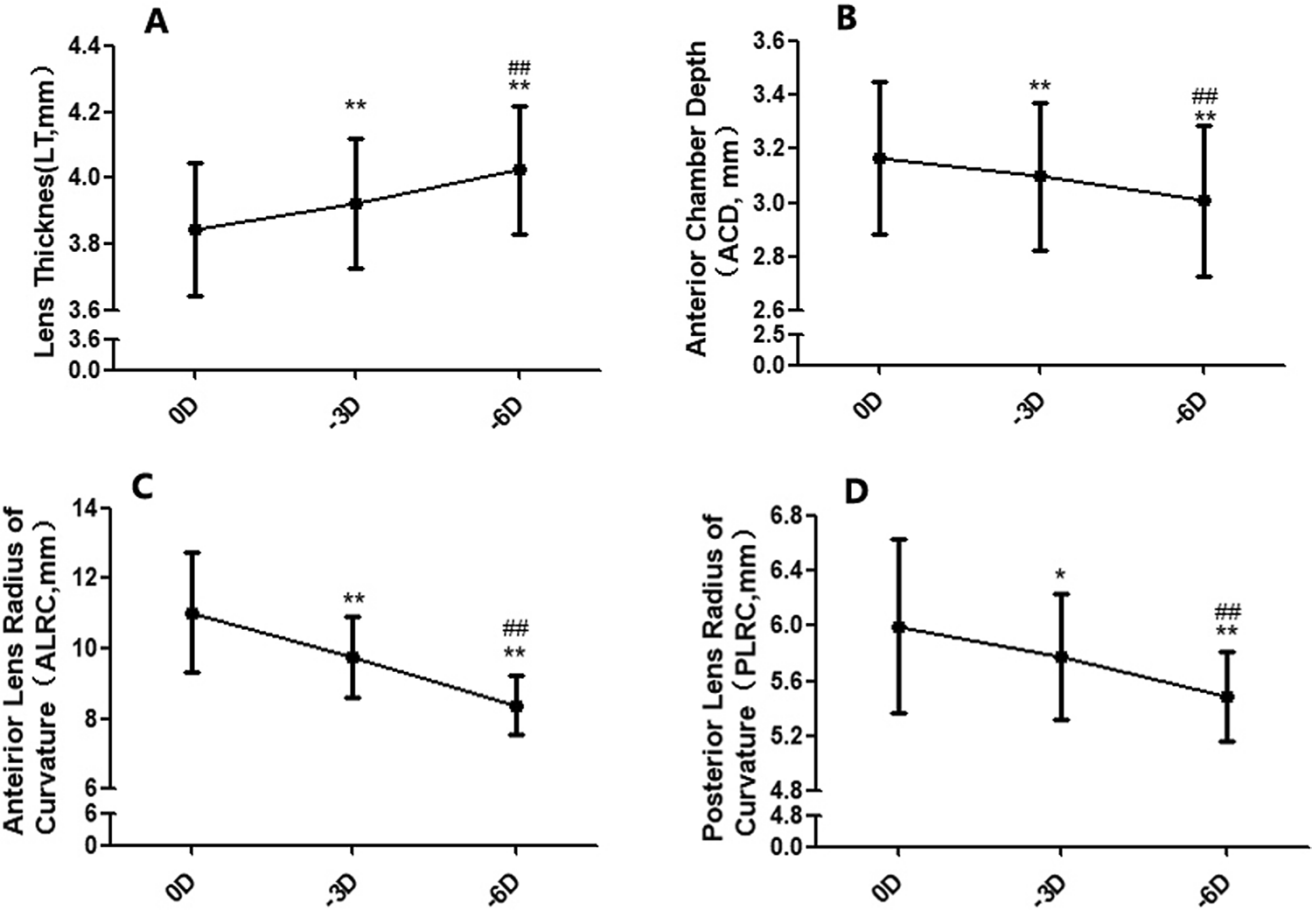
The changes in the lens shape during accommodation. (**A**) The changes of lens thickness (LT). (**B**) The changes of anterior chamber depth (ACD). (**C**) The changes of anterior crystalline lens radius of curvature (ALRC). (**D**) The changes of posterior crystalline lens radius of curvature (PLRC) (compared with 0D, *P < 0.05, **P < 0.01; compared with − 3D, ^##^P < 0.01).

During accommodation, the anterior chamber depth (ACD) became significantly smaller while the lens thickness (LT) significantly increased (ANOVA, F_LT_ = 160.69, P_LT_ = 0.000; F_ACD_ = 118.89, P_ACD_ = 0.000; Fig. 1A,B). With − 6D accommodation stimulation, LT increased from 3.85 ± 0.20 mm to 4.03 ± 0.19 mm. For all subjects, both the anterior and posterior crystalline lens radius of curvature (ALRC and PLRC) became smaller during accommodation: ALRC decreased on average 0.44 mm/D to accommodative stimuli, from 11.02 ± 1.72 mm to 9.75 ± 1.16 mm for − 3D, and to 8.38 ± 0.84 mm for − 6D (F_ALRC_ = 100.01, P_ALRC_ = 0.000, Fig. 1C), PLRC decreased on average 0.09 mm/D, from 6.00 ± 0.63 mm to5.77 ± 0.45 mm for − 3D, and to 5.49 ± 0.32 mm for − 6D (F_PLRC_ = 23.39, P _PLRC_ = 0.000, Fig. 1D).

### The changes of the lens components during accommodation

**Figure 2:**
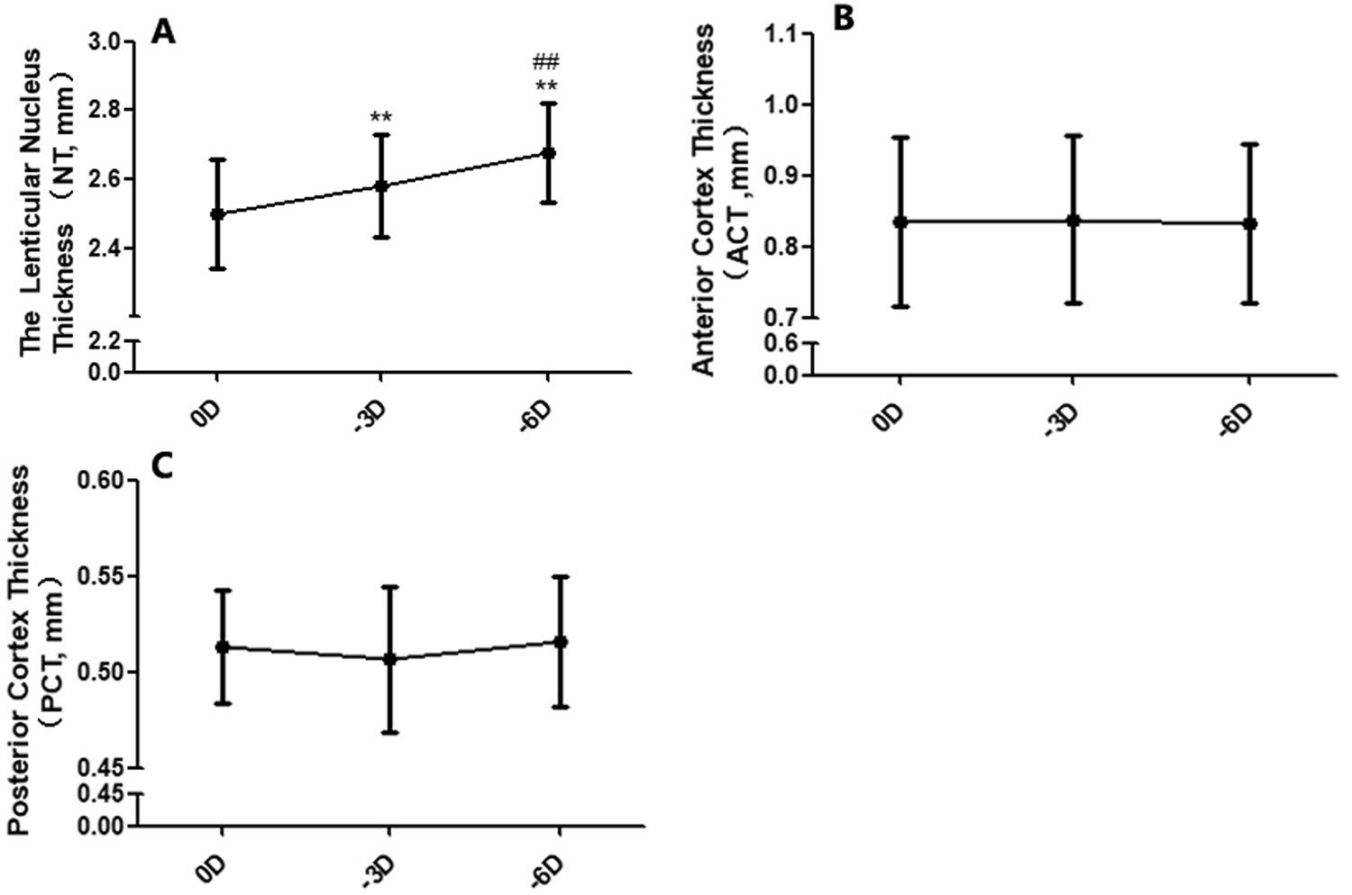
The changes in lens components during accommodation. (**A**) The changes of lenticular nucleus thickness (NT), (**B**) The changes of anterior cortex thickness (ACT), (**C**) The changes of posterior cortex thickness (PCT) (compared with 0D, **P < 0.01; compared with − 3D, ^##^P < 0.01).

**Figure 3:**
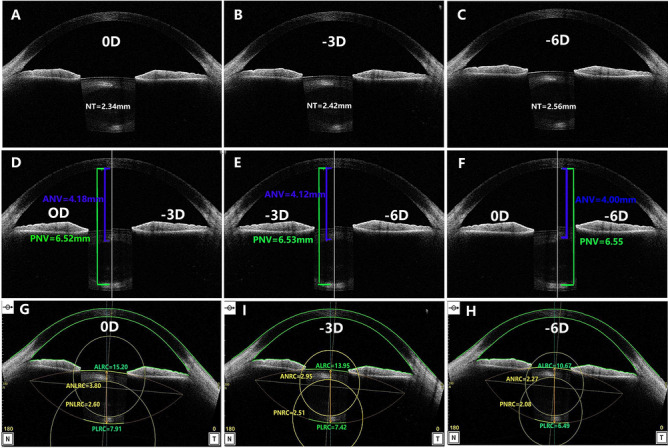
OCT images at different accommodative states in a 35-year-old male with − 1.5D myopia. (**A**–**C**) Graphs show NT in different accommodation states; (**D**–**F**) graphs show ANV and PNV in different accommodation states; (**G**,**H**) graphs show ALRC, PLRC, ANRC and PNRC in different accommodation states (**A**–**F** rotated to show the optical axis vertically, figures were prepared by Yan Xiang with Image-Pro Plus, Version 6.0, MD, USA, https://www.mediacy.com).

In the resting state eye, the average nucleus thickness (NT) was 2.50 ± 0.16 mm, the anterior cortex thickness (ACT) was 0.51 ± 0.03 mm and the posterior cortex thickness (PCT) 0.84 ± 0.12 mm. When accommodation stimulation was given, the NT increased to 2.57 ± 0.15 mm under − 3D and to 2.68 ± 0.14 mm under − 6D stimulation, with an average of 30 μm/D to accommodative stimuli (23 μm/D for 0 to − 3D and 37 μm/D for − 3 to − 6D, F_NT_ = 92.71, P = 0.000, Fig. 2A). There was no difference of ACT and PCT between different accommodation states (F_ACT_ = 0.42, P_ACT_ = 0.659; F_PCT_ = 2.73, P_PCT_ = 0.077, Fig. 2B,C). Representative OCT images for these changes under different accommodative states are shown in Fig. 3 (from a 35-year-old male with − 1.5D myopia).

### The changes in lenticular nucleus curvature and position during accommodation

**Figure 4:**
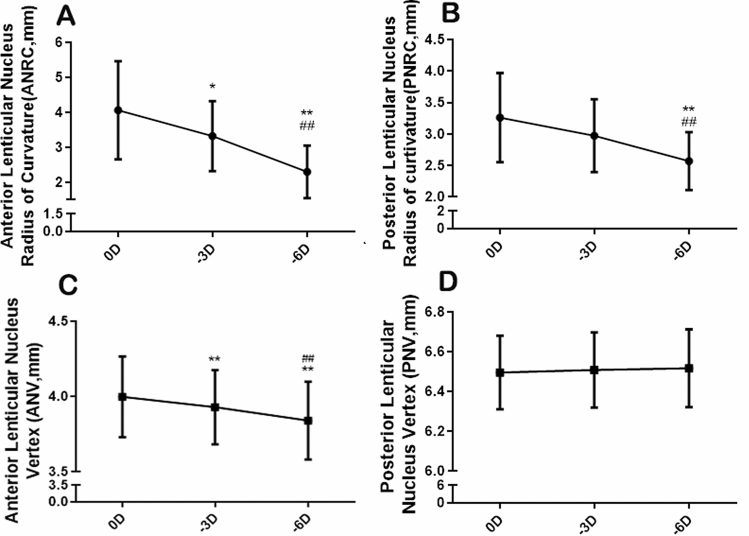
The changes in the lenticular nucleus during accommodation. (**A**) The changes of anterior lenticular nucleus radius of the curvature (ANRC). (**B**) The changes of posterior lenticular nucleus radius of the curvature (PNRC). (**C**) The changes of anterior lenticular nucleus vertex (ANV). (**D**) The changes of posterior lenticular nucleus vertex (PNV) (compared with 0D, *P < 0.05, **P < 0.01; compared with − 3D, ^##^P < 0.01).

In the resting state eye, the average anterior lenticular nucleus radius of the curvature (ANRC) ranged from 2.53 to 8.1 mm (on average 4.06 ± 1.40 mm) while the posterior lenticular nucleus radius of the curvature (PNRC) ranged from 2.26 to 4.67 mm (on average 3.26 ± 0.71 mm). When accommodation stimulation was given, both the ANRC and PNRC clearly decreased (F_ANRC_ = 58.25, P_ANRC_ = 0.000; F_PNRC_ = 19.75, P_PNRC_ = 0.000, Fig. 4A,B). The ANRC decreased to 3.32 ± 1.00 mm for − 3D stimulation and to 2.30 ± 0.75 mm for − 6D, at a speed of 212 μm/D related to accommodative stimuli. In addition, the PNRC decreased to 2.97 ± 0.58 mm for − 3D stimulation and to 2.57 ± 0.46 mm for − 6D, at a speed of 115 μm/D. To investigate displacement of the nucleus, we measured the anterior and posterior lenticular nucleus vertex (ANV and PNV). The ANV significantly moved forward (F_ANV_ = 107.28, P_ANV_ = 0.000, Fig. 4C), which changed from 4.00 ± 0.27 mm to 3.93 ± 0.25 mm for − 3D, and 3.84 ± 0.26 mm for − 6D. However, there was no difference between different accommodation states for the PNV movement (F_PNV_ = 1.54, P_PNV_ = 0.231 Fig. 4D).

## Discussion

In this study we assessed changes in the lens internal components during accommodation in vivo using the CASIA2 OCT. Measuring the exact changes in the human crystalline lens during accommodation is very important in order to understand the mechanism of presbyopia. This is also crucial when designing and evaluating solutions for presbyopia, in particular the lens-based procedures.

Our study revealed the changing pattern of the lens inner components under accommodation stimulation: the lens thickness increment mainly contributed to the lenticular nucleus, but not the cortex. This is line with previous studies. However, the change value in cortex and nucleus varied considerably among researchers due to use of different techniques. Patnaik^6^ firstly studied the component change during accommodation using the slit-lamp photograph technique . He reported about 6% of lens changes in NT and only 0.5% of lens changes in the cortical zones under − 5–7D stimulation demand, but without the exact values reported. Later, Brown^7^ tested 5 cases and reported that the NT increased 0.07 mm/D with − 6D accommodation stimulation in a 29 year old subject, while the posterior cortex slightly increased. By using Scheimpflug slit-lamp photography, Koretz^8^ found an increase of 0.041 mm/D for the NT, 0.002 mm/D for the ACT, and 0.000 mm/D for the PCT under − 2D accommodation stimulation. By deploying the Scheimpflug images technique, with correction made for the distortion due to the geometry of the Scheimpflug imaging system, Dubbelman et al.^9^ demonstrated an increase of 0.046 mm/D for the NT, only − 0.001 mm/D for the ACT, and − 0.002 mm/D for the PCT under − 6D accommodation stimulation. Later utilizing the same technique, they reported on average 0.04 mm/D change for nucleus with accommodation in 5 young people^15^. In our study by using OCT, we only detected 0.03 mm/D for the NT under − 6D stimulation and both the ACT and PCT did not change significantly. In addition, those differences could not only be from different techniques deployed, but other contributors could also be age, race, and accommodation demand vs response. For example, with the OCT, Martinez-Enriquez^14^ also tested the change in ALRC and LT under accommodation stimulation. However, the change amplitude is different to ours which were lower (ALRC − 0.6 mm/D vs − 0.44 mm/D; LT 0.069 mm/D vs 0.03 mm/D).

Previous study showed that the nucleus becomes more convex in morphology during accommodation^15^. In our study by using CASIA2 OCT to measure the nucleus, the surface curvature and position were tested under different accommodative stimulation states. We found that: the ANRC decreased much more than that of PNRC; and the anterior nucleus surface moved forward significantly, but the posterior nucleus surface did not move under accommodation. This indicated that the nucleus changed non-uniformly under accommodative stimulation. We speculate that reasons for a non-uniform change of the nucleus under accommodative stimulation are as following. The human lens continues to grow throughout life, due to the addition of new lens fibers, which gradually push away old fibers, which harden into the nucleus of the lens^16,17^.While, Lens fibers from the anterior cortex are about 3 to 2.4 times greater than those of the posterior cortex^9,18^, as a result, the anterior nucleus possibly less stiff than the posterior nucleus could easily deform during accommodation. Second, the asymmetry distribution of Zonular fibers (anterior, equatorial and posterior suspensory ligament) between the anterior and posterior part of the lens^19^, could result in uniform stretching force and express in conformity mechanical changes when accommodation induced ciliary muscle contraction^20^. In one word, these results indicate that the lenticular nucleus plays a key role in accommodation. With age, the crystal nucleus hardens and loses its response to accommodation and eventually causes the development of presbyopia. Therefore, the lenticular nucleus should be the primary target for accommodation restoration strategies of lens-based procedures for presbyopia. Recently developed techniques such as lens photodisruption or component-based lens refilling may be potential presbyopia correction techniques. It has been reported that lens photodisruption with the femtosecond laser can improve lens elasticity^1,21–23^ , but is limited by the ability to recover accommodation. In future, the strategy could preferentially be to directly reduce the stiffness of the nucleus of the older lens through refining laser patterns and pulse energies, which will achieve more effectively accommodation restoration in presbyopia. Another technique is the component-based lens refilling. The anterior curvature of the lens nucleus changes more than the posterior part under accommodation. To reach similar morphological changes under accommodation, the design strategy should somehow mimic the lens property with gradient refractive index or material stiffness. Thus, possibly achieve phycological re-construction of the lens and restore accommodation in presbyopia.

A major limitation of this study is that all included volunteers were healthy and with a relatively narrow age range of 30–40 years. As accommodation ability usually decreases with age, the changing pattern of the lens inner components under accommodation with age needs to be further studied. Another limitation is that we calculated lens components changes based on accommodative stimulus values, but not subjective accommodative responses. The most accurate way would be to use accommodative responses taken simultaneously with the image capture. The reason is that those factors such as age, race, accommodation demand vs response could contribute to variations in results.

In conclusion, when under accommodation stimulation, lens thickness changed mainly due to the lenticular nucleus, but not the cortex. For the lenticular nucleus, the ANRC decreased more than the PNRC and the nucleus became convex. Further, the anterior surface of the nucleus moved forward while the posterior surface of the nucleus moved backward but only slightly.

## Methods

### Subjects

Twenty-three healthy adults from Tongji community were recruited and testing was performed in Tongji hospital outpatient central. No subjects had any abnormal ocular findings, or any history of ocular diseases, surgery, trauma, or contactlens. Subjects were excluded when the best corrected visual acuity in each eye was lower than 20/20, and the amplitude of accommodation less than − 6D. This study was approved by the research review board of Huazhong University of Science and Technology and the study protocol registered with chictr.org.cn (ChiCTR-ROC-16008832). Informed consent was obtained from each subject, and they were all treated in accordance with the tenets of the Declaration of Helsinki.

### Experimental procedure

Serial regular ocular examinations were performed to screen subjects with ocular diseases other than refractive error: these include slit lamp, fundus examination, intraocular pressure (IOP) and subjective optometry. Afterwards, the amplitude of accommodation was measured using the minus lens test as reported by León^24^ and subjects were excluded if their accommodation amplitude was less than − 6D. Subjects were then asked to undergo an OCT test in different accommodation stimuli.

### OCT image

OCT examination was performed under a standard procedure with a swept-source OCT (CASIA2; Tomey Corporation, Nagoya, Japan) in the morning (9:00 a.m.–11:00 a.m.). To avoid head movement between different scans, subjects were asked to hold their jaw and forehead onto the fixed trestle, stare at the optotype with the testing eye during scanning. The location of the machine was locked during testing. All OCT images were obtained in the same examination room with controlled environmental settings of temperature (15–25 °C) and humidity (30–50%) and the light was dimmed to avoid possible pupillary constriction. Before scanning, the refractive error was corrected with a built-in program. Different accommodation states were achieved by a built-in program and subjects were asked to clearly look forward at an internal fixation target symbol “ ”. The lens analysis mode (Accommodation load, Starburst target.) was used to capture images of the anterior segment of the eye. Pictures were taken when the subject reported a clear view of the target symbol for 5 s at different accommodation states in sequence organized as follows: 0D, − 3D and − 6D accommodation stimuli.

### Image analysis

**Figure 5:**
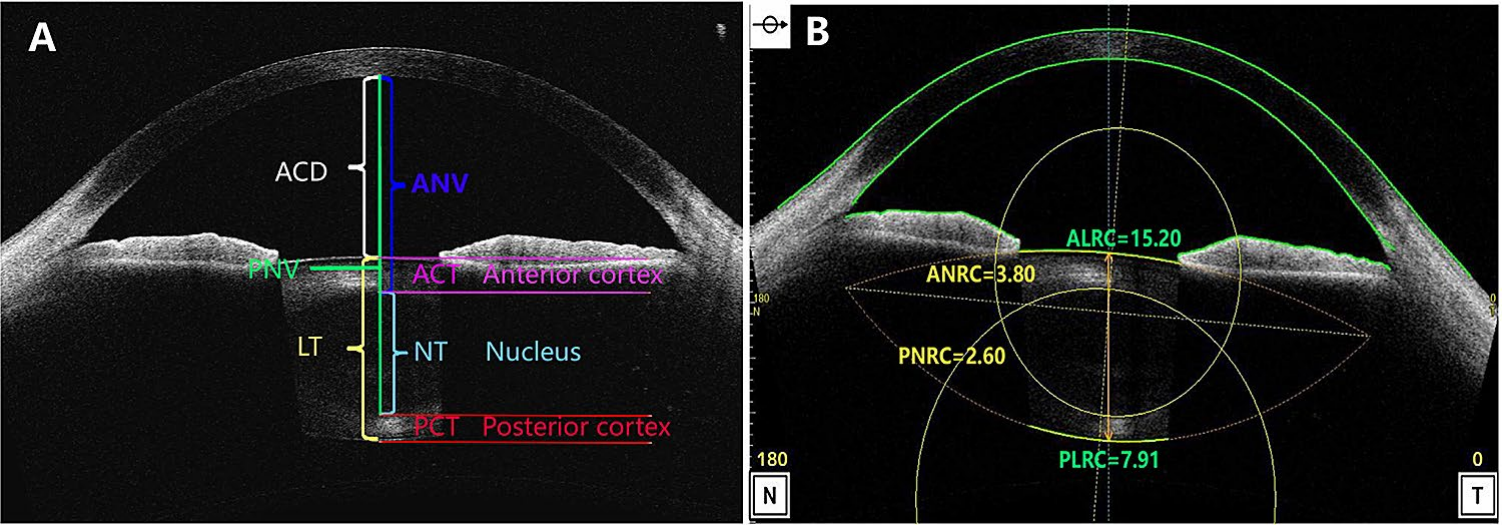
Examples of measured items and methods in CASIA2 optical coherence tomography (OCT) image. (**A**) Measured items: Anterior chamber depth (ACD), Lens thickness (LT), Lenticular nucleus thickness(NT), Lenticular cortex thickness (CT), Anterior cortex thickness (ACT), Posterior cortex thickness (PCT), Anterior lenticular nucleus vertex (ANV), Posterior lenticular nucleus vertex (PNV). (**B**) Showing measurement methods for the anterior crystalline lens radius of the curvature (ALRC, green) and the posterior crystalline lens radius of the curvature (PLRC, green). The anterior crystalline lenticular nucleus radius of the curvature (ANRC, yellow arc), the posterior crystalline lenticular nucleus radius of the curvature (PNRC, yellow arc). (**A** rotated to show the optical axis vertically, figures were prepared by Yan Xiang with Image-Pro Plus, Version 6.0, MD, USA, https://www.mediacy.com).

The CASIA2 enables some automatic measurements. Anterior segment parameter measurements, including ATA, CT, ACD, ALRC and PLRC, were obtained from images by the built-in software. The LT, ACT, PCT, NT, ANRC, PNRC, ANV and PNV in each image were quantified manually and measured using the Image-pro plus software (Version 6.0, MD, USA, https://www.mediacy.com/). Measuring items were determined based on two-dimensional images (examples demonstrated in Fig. 5). The anatomical details of the lens such as the capsule, cortex and nucleus can easily be distinguished and identified (Fig. 5A). The anterior and posterior interfaces of the lenticular nucleus were segmented using edge detection with the tool of “Fit circle”. The lenticular nucleus thickness (NT) defined in this study was equivalent to the distance between the C3 zones base on the Oxford system^25,26^*.* The ANRC and PNRC were measured by manually depicting 3 points surrounding the outline of the anterior and posterior surface of the nucleus. Then the ANRC and PNRC were segmented and calculated utilizing this mi-automated fitting method with two elliptic paraboloid surfaces using the best fit arc feature with the Image-pro plus software (Fig. 5B).

### Quality control

Researchers were trained before conducting the study. OCT scanning were performed by a skilled operator. The scan was taken once for each accommodation status and three times in total. The ambient lighting conditions were kept constant during the whole procedure to avoid significant variations in pupil diameter. All measurement items were sequentially measured under three different accommodation conditions (0D, − 3D, − 6D accommodation). As we did before, the images of these eyes were analyzed by two observers who were blinded to treatments, the intraobserver reproducibility and interobserver reproducibility were also evaluated^27^. Only those testing items whose intraclass correlation coefficient value is not less than 0.75 will be presented.

### Statistical analysis

Data were analyzed using SPSS 19.0 (IBM Corp., Armonk, NY, USA). The sample size was calculated by assuming that there is a difference in lens thickness between different accommodation states, for repeated measures analysis of variance (rANOVA) with a correlation among repeated measures with a value of 0.8. A medial level of partial eta square of 0.06 was adopted, which gave an effect size of about 0.25. A sample size of at least 19 participants was deemed to be sufficient to give us a power of 0.80 with 95% confidence. The final sample size was adjusted to 23 based on the 20% participant loss. Quantitative data are presented as mean ± standard deviation. Repeated measure ANOVA was performed to reveal significant differences among different accommodation states. Prior to the repeated measure ANOVA, the sphericity assumption was checked using the Mauchly’s sphericity test. And when the sphericity test was not statistically significant, the Greenhouse–Geisser correction was applied. The Bonferroni procedure was used as a post hoc test for comparisons between groups. P < 0.05 was set as statistical significance in all cases.
